# Transcriptional Regulation of Autophagy: Mechanisms and Diseases

**DOI:** 10.3389/fcell.2019.00114

**Published:** 2019-07-02

**Authors:** Chiara Di Malta, Laura Cinque, Carmine Settembre

**Affiliations:** ^1^Telethon Institute of Genetics and Medicine, Pozzuoli, Italy; ^2^Department of Medical and Translational Sciences, University of Naples Federico II, Naples, Italy

**Keywords:** autophagy, TFEB, genetic diseases, nucleus, transcription, lysosomal storage disease

## Abstract

Macro (Autophagy) is a catabolic process that relies on the cooperative function of two organelles: the lysosome and the autophagosome. The recent discovery of a transcriptional gene network that co-regulates the biogenesis and function of these two organelles, and the identification of transcription factors, miRNAs and epigenetic regulators of autophagy, demonstrated that this catabolic process is controlled by both transcriptional and post-transcriptional mechanisms. In this review article, we discuss the nuclear events that control autophagy, focusing particularly on the role of the MiT/TFE transcription factor family. In addition, we will discuss evidence suggesting that the transcriptional regulation of autophagy could be targeted for the treatment of human genetic diseases, such as lysosomal storage disorders (LSDs) and neurodegeneration.

## Introduction

Autophagy is an evolutionary conserved catabolic process devoted to the degradation of intracellular components. Three main types of autophagy have been described to date: macroautophagy, microautophagy, and chaperon-mediated autophagy. Macroautophagy involves the formation of a double-membrane vesicle, the autophagosome, which captures cytoplasmic contents and then fuses with lysosomes to generate autophagolysosomes, structures in which cargo substrates are degraded by lysosomal enzymes (Mizushima et al., 2008; He and Klionsky, 2009; Hurley and Schulman, 2014). In microautophagy, cytoplasmic constituents are directly imported into the lysosome and degraded (Ahlberg et al., 1982; Mijaljica et al., 2011; Sahu et al., 2011), while chaperon-mediated autophagy is characterized by the translocation of cytosolic proteins harboring the pentapeptide KFERQ sequence across the lysosomal membrane for degradation (Kaushik and Cuervo, 2012). Thus, the three types of autophagy rely on functional lysosomes to digest intracellular cargos.

Macroautophagy (herein referred to as autophagy) is constitutively active, albeit at low levels, in most cells of our body as part of the constitutive turnover of cytosolic components (Mizushima and Komatsu, 2011). This is generally referred as “basal autophagy.” In addition, different cellular stimuli, in particular nutrient starvation, can potently stimulate autophagy to enhance the degradation of cytosolic components to generate energy (Kaur and Debnath, 2015). Two nutrient-responsive kinases, mTORC1 and AMPK, rapidly respond to nutrient fluctuations and phosphorylate critical regulators of autophagosome biogenesis and maturation (e.g., fusion with lysosomes) (Egan et al., 2011). In particular, in the presence of nutrients, mTORC1 phosphorylates two fundamental autophagy initiation proteins, unc-51-like autophagy activating kinase (ULK)1 and ATG13, inhibiting their pro-autophagic activity (Hosokawa et al., 2009). Conversely, nutrient depletion inactivates mTORC1 and concomitantly activates AMPK, which phosphorylates ULK1 and ATG13 on specific amino acid residues promoting ULK1/ATG13 complex activity and autophagy initiation (Shang et al., 2011). In addition, several other mechanisms of post-translational regulation of autophagy in response to nutrient fluctuations have been described and reviewed elsewhere [see for example reviews (He and Klionsky, 2009; Kuma and Mizushima, 2010; Rabinowitz and White, 2010; Mizushima et al., 2011)].

The modulation of autophagy in the maintenance of cellular homeostasis goes far beyond the response to nutrient fluctuation, as cells exploit autophagy to eliminate damaged organelles, misfolded proteins, and invading organisms (Deretic et al., 2006; Mizushima et al., 2008). Deregulation of these autophagy-dependent cytoprotective functions has been associated to different pathologies, including immune disorders, neurodegenerative diseases, cancer and aging (Deretic et al., 2006; Hara et al., 2006; Komatsu et al., 2006; Harris and Rubinsztein, 2011; Mizushima and Komatsu, 2011; White, 2015).

For a long time, autophagy was considered as a pathway exclusively regulated by cytosolic processes. This concept was supported by the observation that enucleated cells still form autophagosomes (Morselli et al., 2011). However, increasing amounts of evidence collected in the last decade clearly indicate that nuclear transcriptional and epigenetic events play a major role in autophagy regulation. This review aims to summarize the “nuclear” control of autophagy, focusing in particular on the co-regulation of autophagy and lysosome biogenesis by the transcription factor EB (TFEB).

## Transcriptional Regulation of Autophagy

The first observation that autophagy can be induced at the transcriptional level was made in yeast in Kirisako et al. (1999), who reported that nitrogen starvation induced the upregulation of the essential autophagy gene Apg8p, the homologous of mammalian LC3. In the last 10 years several laboratories demonstrated that transcription factors that enhance the expression of autophagy genes (even few of them) increase autophagy and the degradation of unwanted substrates [see below and (Lapierre et al., 2015; Füllgrabe et al., 2016)]. These observations opened a new, unexpected, scenario indicating that autophagy activity could in fact be modulated from the nucleus.

## TFEB and MiT Factors

Transcription factor EB is a member of the microphthalmia/ transcription factor E (MiT/TFE) family of transcription factors (TFs) that also includes MITF, TFE3, and TFEC proteins (Hemesath et al., 1994). They belong to the larger family of basic helix-loop-helix leucine zipper (bHLH-Zip) transcription factors, such as MYC, MAD, and MAX, and share a basic DNA-binding domain, and an HLH plus a leucine zipper domain important for dimerization (Beckmann et al., 1990; Sato et al., 1997; Steingrimsson et al., 2002). The homo- or hetero- dimerization is necessary to activate transcription. MiT/TFE members can only form heterodimers among each other due to structural constraints in their leucine zipper domain (Hemesath et al., 1994; Pogenberg et al., 2012). Binding to DNA is mediated by the recognition of a common DNA hexanucleotide sequence (CACGTG) known as the E-box (Hemesath et al., 1994). This sequence conforms to the canonical CANNTG motif, recognized by other bHLH-Zip transcription factors, however, specific nucleotide residues that flank this motif characterize the coordinated lysosomal expression and regulation (CLEAR) motif (GTCACGTGAC) that is preferentially recognized by MiT/TFE members (Sardiello et al., 2009; Palmieri et al., 2011; Martina et al., 2014). Bioinformatic analysis identified one or more CLEAR motifs in the promoter region of many lysosomal genes. Notably, these genes belong to different functional lysosomal categories, (ion channels, hydrolases, and transmembrane proteins, etc.) so that TFEB activation leads to a global enhancement of lysosomal catabolic efficiency (Sardiello et al., 2009).

In addition, TFEB also regulates the expression of genes involved in different steps of the autophagy process, such as genes important for autophagy initiation (*BECN1, WIPI1, ATG9B*, and *NRBF2*) autophagosome membrane elongation (*GABARAP, MAP1LC3B*, and *ATG5*), but also genes important for substrate capture (*SQSTM1*) and for autophagosomes trafficking and fusion with lysosomes (*UVRAG, RAB7*) (Palmieri et al., 2011; Settembre et al., 2011). As a result, TFEB activation induces a striking increase in autophagy flux. Similarly, TFE3 and MITF were successively identified as regulators of autophagy and lysosomal biogenesis (Martina et al., 2014; Ploper et al., 2015).

Transcription factor EB activity is largely controlled by its subcellular localization, which is mainly regulated by phosphorylation (Puertollano et al., 2018). Phosphorylated TFEB is sequestered into the cytosol, hence the transcriptional induction of its target genes is inhibited. Conversely, upon nutrient starvation, TFEB is dephosphorylated and rapidly translocates into the nucleus where it binds to the promoter of target genes (Settembre et al., 2011). To date, different kinases that phosphorylate TFEB have been identified. mTOR, as part of the protein complex mTORC1, represents the main kinase responsible for TFEB phosphorylation in presence of amino acids (Peña-Llopis et al., 2011; Martina et al., 2012; Roczniak-Ferguson et al., 2012; Settembre et al., 2012). Inhibition of TFEB activity via phosphorylation of conserved amino acid residues (Ser 142, Ser 211, Ser122, and Ser138) is part of a larger metabolic response mediated by mTORC1 aimed to shut-off catabolic pathways while turning on anabolic ones when nutrients are available (Martina et al., 2012; Roczniak-Ferguson et al., 2012; Settembre et al., 2012; Vega-Rubin-de-Celis et al., 2017; Napolitano G. et al., 2018). Similarly, mTORC1 also regulates the nuclear localization of TFE3 and some isoforms of MITF, thus efficiently inhibiting transcriptional induction of lysosome biogenesis and autophagy (Martina et al., 2014).

**FIGURE 1:**
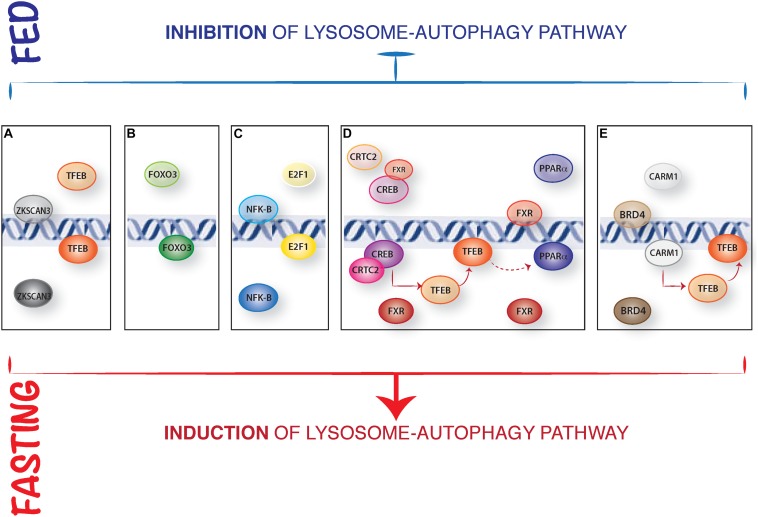
Representative model of the nuclear control of lysosome-autophagy pathway. **(A)** Opposed regulation of ZKSCAN3 and TFEB. In presence of nutrients, TFEB is cytosolic, and the transcription factor ZKSCAN3 localizes in the nucleus, inhibiting lysosome gene expression. During starvation, ZKSCAN3 translocates into the cytosol and TFEB translocates into the nucleus where activates lysosome-autophagy gene expression. **(B)** The nuclear translocation and activation of FOXOs transcription factors is induced IN serum starved condition. **(C)** NFKB binds to the promoter and represses Bnip3 expression in fed conditions, while during starvation Bnip3 expression is promoted by E2F1. **(D)** In presence of nutrients, FXR inhibits autophagy by preventing the binding of PPARα to DNA and by inhibiting CREB interaction with its coactivator CRTC2. Conversely, during starvation FXR activity is inhibited, and CREB-CRTC2 complex is formed and binds to the promoters of lysosomal autophagy genes and of TFEB; similarly, starvation-mediated inhibition of FXR allows PPARα binding to the DR1 elements in the promoters of autophagy genes. **(E)** Epigenetic regulation of autophagy: in fed status, CARM1 is inactive and BRD4 represses the expression of autophagic and lysosomal genes regulating Histone3 lysine9 methylation. In fast state, BRD4 is inactive and CARM1 translocates into the nucleus promoting lysosomal-autophagy gene expression via a positive Histone3 Arginine17 methylation and inducing TFEB transcriptional activity.

In addition, mTORC1 can inhibit TFEB transcriptional activity by modulating the zinc finger transcription factors harboring Kruppel-associated box (KRAB) and SCAN domain (ZKSCAN3) activity (Chauhan et al., 2013). ZKSCAN3 represses a large group of lysosomal and autophagy genes when nutrients, in particular amino acids, are present in the cell. Conversely, treatment with the mTOR inhibitor Torin1 induced ZKSCAN3 nuclear exclusion. Silencing of ZKSCAN3 augmented TFEB-mediated lysosomal and autophagic activation suggesting that these two transcription factors act in opposite ways to regulate autophagy in response to nutrient fluctuations (Figure 1A). While this mechanism appears to be relevant in cell culture experiments, its relevance *in vivo* is unclear (Pan et al., 2017).

In addition to mTORC1, other growth-regulating kinases control TFEB nuclear localization. ERK2 was the first kinase to be associated with TFEB phosphorylation in response to nutrients availability (Settembre et al., 2011). In particular, ERK2 mediated phosphorylation of TFEB at Ser142 inhibited TFEB nuclear translocation thus limiting transcriptional activation of its downstream target genes (Settembre et al., 2011; Li et al., 2018, 2019). Subsequently, the glycogen synthase kinase 3 beta (GSK3B) was identified as the kinase responsible for TFEB phosphorylation at Ser134 and 138 (Li et al., 2016). This event, coupled to phosphorylation at Ser142 by ERK2 and mTORC1, unmasks a nuclear export localization signal required for TFEB cytosolic accumulation (Li et al., 2018). Moreover, the Akt and the PKCβ kinases phosphorylate TFEB at c-terminal critical serines, but this phosphorylation seems to control TFEB stability rather than its nuclear localization (Ferron et al., 2013; Palmieri et al., 2017).

Transcription factor EB nuclear translocation can also be triggered by activation of the calcium and calmodulin dependent serine/threonine phosphatase calcineurin (Medina et al., 2015). Notably, the calcium efflux through the lysosomal cation channel Mucolipin1 triggers calcineurin-mediated TFEB dephosphorylation and activation, hence providing a mechanistic explanation of autophagy regulation by calcium signaling.

More recently, the protein phosphatase 2A (PP2A) has been shown to dephosphorylate TFEB upon induction of acute oxidative stress by sodium arsenite (Martina and Puertollano, 2018).

To date, the mechanisms controlling TFEB nuclear export are less characterized but seem to be dependent on the CRM1 exportin and on the presence of a TFEB nuclear export sequence (Napolitano G. et al., 2018). Intriguingly, mTOR-dependent TFEB re-phosphorylation in the nucleus seems to play a major role in TFEB nuclear export.

These studies indicate that several signaling events regulate TFEB subcellular localization, thus placing the transcriptional activation of the lysosomal-autophagy pathway as a general response to cope with different types of cellular stresses.

## FOXO Factors

The class O of forkhead box transcription factors (FOXO) family has an established role in autophagy regulation (Webb and Brunet, 2014). In mammals, this family includes four members: FOXO1, FOXO3, FOXO4, and FOXO6. The activity of three out of four members (FOXO1, FOXO3, and FOXO4) is mainly regulated by AKT phosphorylation in response to growth factors and insulin stimulation. FOXO3 was the first FOXO member identified as a transcriptional regulator of several autophagy genes (*ATG4, ATG12, BECN1, BNIP3, LC3, ULK1, ULK2*, and *VPS34*) in muscle (Mammucari et al., 2007; Zhao et al., 2007; Sanchez et al., 2012). Similar to what reported for MiT/TFE family of transcription factors, FOXO3 transcriptional activity is mostly regulated by a nuclear/cytosolic shuttling. Once activated by growth factors, AKT phosphorylates FOXO3 and this results in its cytoplasmic retention, thus inhibiting transcriptional activation of its target genes. Later on, another member of this family, FOXO1, was also described as a transcriptional regulator of different autophagy genes (Liu et al., 2009; Xu et al., 2011; Xiong et al., 2012). However, FOXO1 also induces autophagy in a transcriptional-independent way: in response to oxidative stress or serum starvation, FOXO1 is acetylated in the cytosol and binds to Atg7 thus favoring autophagy induction by direct interaction with key regulators of autophagosome biogenesis (Zhao et al., 2010; Figure 1B). More recently FOXO transcription factors have been shown to cooperatively control autophagy in cartilage and protect against osteoarthritis (Matsuzaki et al., 2018).

Most notably, a study using *Caenorhabditis elegans* demonstrated that DAF16 (FOXO in mammals) physically and functionally cooperates with HLH30 (TFEB in mammals) to ensure appropriate expression of target genes during organismal responses to stressors (Lin et al., 2018). It will be important to understand whether a FOXO-TFEB cooperation occurs also in mammals.

## P53

Different studies suggest that P53, the most studied tumor suppressor protein, is an inducer of the autophagy pathway. P53 was initially described to promote autophagy by inhibiting the mTORC1 pathway, through transcriptional induction of Sestrin proteins, which activate AMPK while inhibiting mTORC1 lysosomal recruitment (Budanov and Karin, 2008; Chantranupong et al., 2014), and by inducing the expression of the Damaged-regulated- modulator DRAM, a lysosomal protein, which induces autophagy through a yet not identified mechanism (Crighton et al., 2006). Subsequently, a combined CHIP-SEQ and RNA-SEQ analysis performed on mouse embryonic fibroblasts (MEFs) upon DNA-damage, revealed that P53 controls the expression of several genes essential for autophagy induction (*LKB1, ULK1/2*), and autophagosome maturation (*ATG4, ATG7*, and *ATG10*) (Kenzelmann Broz et al., 2013). Moreover, P53 regulates both FOXO3a expression and activity (You et al., 2004; Fu et al., 2009; Miyaguchi et al., 2009; Renault et al., 2011), and promotes TFEB/TFE3 nuclear translocation upon DNA damage (Jeong et al., 2018), thus controlling key upstream modulators of the autophagy pathway.

However, cytoplasmatic P53 may also act as a negative regulator of autophagy, although the mechanisms underlying this inhibitory regulation are still elusive (Green and Kroemer, 2009; Comel et al., 2014). Further studies are needed to fully define the role of P53 in the regulation of autophagy pathway.

## E2F1/NF-kB Axis

The transcription factors E2F1 and NF-kB regulate autophagy through the regulation of BNIP3 expression (Tracy et al., 2007; Gang et al., 2011). BNIP3 is a hypoxia-induced activator of autophagy that disrupts the inhibitory binding of B-cell lymphoma 2 (BCL-2) to Beclin1, a component of the class III phosphatidylinositol-3-OH kinase (PI3K) complex, that promotes autophagosome biogenesis. During normoxia, NF-kB constitutively binds to the promoter of BNIP3 repressing its expression (Shaw et al., 2008). Hypoxia reduces the occupancy of NF-kB on the BNIP3 promoter thus allowing E2F1 to induce its expression and activate autophagy (Figure 1C). In addition, E2F1 can also promote the expression of other autophagy genes, such as *ULK1, LC3*, and *ATG5* (Polager et al., 2008).

## CREB-FXR and PPARα-FXR Circuits

The farnesoid X receptor (FXR) represses liver autophagy during feeding conditions (Thomas et al., 2008; Calkin and Tontonoz, 2012). FXR is activated by increased bile acid levels after feeding and transcriptionally represses several autophagy genes through two apparently independent mechanisms. Seok et al. (2014) proposed that FXR inhibits the transcriptional activity of the fasting-activated cAMP response element-binding protein (CREB) by impeding the interaction between CREB and its coactivator CRTC2. Upon fasting, FXR inhibition is relieved thus allowing the CREB-CRTC2 complex to form and induce the expression of many autophagy genes, including *ATG7, ULK1*, and *TFEB* (Figure 1D). Interestingly, TFEB also regulates the expression of genes important for lipid metabolism in the liver, suggesting that its role in the FXR-CREB axis might be not limited to autophagy regulation (Settembre et al., 2013). In addition, Lee et al. (2014) identified the nuclear receptor Peroxisome Proliferator Activated Receptor alpha (PPARα) as the transcriptional activator that opposes FXR in response to nutrient availability. FXR and PPARα share the ability to bind to specific DNA sites (DR1 elements) in the promoter regions of many autophagy-related genes, so that these two nuclear receptors compete for the binding to the same target genes. Fasting activates PPARα while inhibiting FXR, thus inducing transcriptional activation of autophagy genes in liver (Figure 1D). Notably, TFEB transcriptionally enhances the expression of *PPARα* and its coactivator peroxisome proliferator activated receptor gamma 1 alpha (*PGC1α*) (Settembre et al., 2013), suggesting that the induction of *TFEB* expression by CREB could in turn potentiate PPARα activity. Thus, it is possible that both the FXR-CREB and FXR-PPARα circuits coexist and participate to the coordination of autophagy with other metabolic processes (e.g., lipid degradation) occurring in the liver.

## Epigenetic Regulation of Autophagy

Histone post-translational modifications, such as methylation, acetylation, and deacetylation, influence the overall chromatin structure, thus affecting the accessibility of transcription factors to chromatin (Lawrence et al., 2016). To date, several examples of epigenetic regulations of the autophagy pathway have been described.

### Histone Methylation

The epigenetic reader Bromodomain-containing protein 4 (BRD4) has been identified as a repressor of a transcriptional program that promotes autophagy and lysosome biogenesis (Sakamaki et al., 2017). In presence of nutrients, BRD4 represses the expression of several autophagic and lysosomal genes by recruiting the histone lysine methyltransferase G9a, which deposits a repressive H3K9diMe in the promoters of lysosomal and autophagy genes. Conversely, nutrient depletion promotes AMPK-mediated BRD4 inhibition and the expression of lysosomal and autophagic genes through a yet-to be characterized transcriptional regulator.

The co-activator-associated arginine-methyltransferase 1 (CARM1) was recently identified as a key autophagy regulator (Shin et al., 2016). Glucose (but also amino acid) starvation leads to a CARM1-dependent increase in histone H3 Arg17 dimethylation levels at the promoters of autophagy and lysosomal genes and this is critical for proper autophagy activation. Mechanistically, upon starvation CARM1 translocates into the nucleus where binds TFEB and promotes the transcriptional activation of its target genes. CARM1 seems to be essential for TFEB-mediated autophagy activation since TFEB overexpression fails to increase autophagy in cells lacking CARM1 (Figure 1E).

### Histone Acetylation

Recently, a global decrease in acetylation levels of H4K16 was described upon nutrient starvation and/or mTOR inhibition (Füllgrabe et al., 2013). This downregulation translates into a transcriptional repression of key autophagy genes in order to prevent a chronic autophagy induction, which could be lethal. These responses are dependent on the histone acetyltransferase hMOF/KAT8/MYST1.

The NAD+-dependent deacetylase Sirt1 regulates autophagy through its deacetylase activity on non-histone cytosolic targets (Lee et al., 2008; Bao and Sack, 2010). Sirt1 may induce autophagy directly by deacetylating autophagy proteins such as ATG5, ATG7 and LC3. Sirt1 might also control the stability of mRNAs encoding for lysosomal enzymes (Latifkar et al., 2019). Moreover, Sirt1 deacetylates the transcriptional regulators of autophagy FOXO1 and FOXO3, enhancing their transcriptional activity (Brunet et al., 2004). Finally, Sirt1 promotes autophagy by activating AMPK, via deacetylation of LKB1 (Lan et al., 2008), while inhibiting mTORC1 signaling favoring its interaction with the TSC1/TSC2 complex (Ghosh et al., 2010).

Additional epigenetic modifications related to autophagy induction are H3K9 methylation (Artal-Martinez de Narvajas et al., 2013), H3K56 acetylation (Chen et al., 2012) and H4K20 methylation (Kourmouli et al., 2004). These are associated with suppression of autophagy, even if further studies are required to clarify their regulation.

## MITF Factors and Human Diseases

The autophagy pathway is important in several processes required to maintain cellular homeostasis, including adaptation to metabolic stress, removal of dangerous cargo, and prevention of DNA damage. If any of these protective functions are impaired, onset and progression of several diseases, such as infection, cancer, neurodegeneration, cardiovascular diseases, and aging may be favored (Mizushima et al., 2008; Harris and Rubinsztein, 2011; Mizushima and Komatsu, 2011; White, 2015). Therefore, it is not surprising that a long list of diseases is associated to mutations in autophagy-related genes [recently reviewed in Levine and Kroemer (2019)]. However, it is important to note that several autophagy proteins participate to other cellular processes, such as vesicular trafficking, phagocytosis, exocytosis, and even cell cycle regulation and immunity, thus the link between disease manifestation and autophagy dysfunction might be difficult to establish (Levine and Kroemer, 2019). This is particularly true for transcription factors, that control the expression of target genes implicated in a number of diverse cellular functions. The activity and/or the localization of TFEB has been reported to be deregulated in several neurodegenerative diseases, such as X-linked spinal and bulbar muscular atrophy (Cortes et al., 2014), Parkinson disease (Decressac et al., 2013), Huntington disease (Tsunemi et al., 2012), and Alzheimer disease (Reddy et al., 2016). These neurodegenerative disorders are characterized by intracellular protein aggregation and autophagy dysfunction, which is predicted to contribute to disease establishment (Menzies et al., 2015). Notably, forced overexpression of TFEB in cellular and murine models of these disorders significantly reduced protein aggregation attenuating pathological manifestation, suggesting that TFEB represents an appealing target for therapy (Sardiello et al., 2009; Dehay et al., 2010; Tsunemi et al., 2012; Decressac et al., 2013; Polito et al., 2014; Xiao et al., 2014, 2015; Chauhan et al., 2015; Kilpatrick et al., 2015).

Lysosomal storage disorders (LSDs) are a class of rare diseases due to mutations in genes encoding for lysosomal proteins (Ballabio and Gieselmann, 2009; Cox and Cachón-González, 2012; Platt et al., 2018). As a consequence, cells show progressive accumulation of indigested material within lysosomes and, eventually, impaired autophagy flux. Interestingly, TFEB was found to be predominantly nuclear in several LSD cellular models (Sardiello et al., 2009; Bartolomeo et al., 2017). The increased nuclear localization of TFEB may be interpreted as an attempt to compensate for the decreased autophagy flux and lysosomal degradative function. While in this context the physiological induction of the TFEB seems to be unable to fully counteract disease progression, TFEB overexpression in different LSDs, such as multiple sulfatase deficiency and mucopolysaccharidosis IIIA (Medina et al., 2011), Pompe disease (Spampanato et al., 2013), Batten disease (Palmieri et al., 2017), Gaucher and Tay Sachs disease (Song et al., 2013), and cystinosis (Rega et al., 2016) resulted effective in reducing lysosomal storage. This effect is most likely the consequence of TFEB’s ability to concomitantly induce lysosomal exocytosis, autophagy and lysosome biogenesis. Similarly, TFEB overexpression in liver had beneficial effects in mouse models of alpha1-antitrypsin deficiency and hepatic hyperammonemia (Pastore et al., 2013; Soria et al., 2018). Notably, by increasing the autophagic degradation of intracellular lipid droplets, TFEB also represents a potential therapeutic target to fight metabolic syndrome associated with obesity (Settembre et al., 2013). Despite the induction of TFEB activity looks as a promising therapeutic tool for several diseases, the side effects of its long-term overexpression must be considered. The over-activation of MiT family of transcription factors is associated with different types of cancer. MITF genomic amplification is frequently found in melanoma, while chromosomal translocations and rearrangements of TFE3 and TFEB are associated with pediatric renal cell carcinomas and alveolar soft part sarcoma (Argani et al., 2001; Haq and Fisher, 2011; Kauffman et al., 2014). Moreover, upregulation of MiT/TFE members has also been observed in pancreatic ductal adenocarcinoma (Perera et al., 2015).

How over-activation of these TFs may favor pro-tumorigenic processes is not completely clear, but recent data indicate that hyper-activation of mTORC1 signaling is a common feature of MiT/TFE associated malignancies (Di Malta et al., 2017). This signaling deregulation depends on the constitutive induction of the essential components of the mTORC1 amino acid sensing machinery RagD and RagC GTPases, direct downstream targets of MiT/TFE TFs. Interestingly, at least in pancreatic ductal adenocarcinoma, the upregulation of MiT/TFE factors leads to simultaneous mTORC1 hyperactivation and autophagy induction and presumably both pathways are exploited by tumor cells to efficiently compete with non-transformed cells (Perera et al., 2015, 2019; Di Malta et al., 2017). In light of the pathological consequences of the constitutive activation of MiT/TFE factors, a pulsatile approach aimed at enhancing TFEB activity only for a certain time-frame could represent a therapeutic strategy for diseases that might benefit of the stimulation of the lysosomal/autophagy pathway.

## Conclusion

In the last years, several studies provided conclusive evidence that autophagy is a transcriptionally regulated process. However, despite different transcriptional modulators of autophagy have been identified, we still know very little about the physiological relevance of this nuclear regulation. The most likely hypothesis is that transcriptional regulation of autophagy cooperates with the post-translational regulation to achieve a fine tuning of autophagy flux particularly in conditions of prolonged starvation or chronic stress. Indeed, the degradation of autophagy proteins, in particular those serving as cargo receptors, is enhanced during autophagy, and similarly lysosomes are utilized during the formation of autolysosomes. Hence, the transcriptional induction of lysosomal and autophagy genes might counteract the depletion of the correspondent proteins during autophagy. Consistently, the translation of mRNAs encoding for proteins with catabolic roles is spared from the general inhibition of protein synthesis during nutrient starvation (Saikia et al., 2016). Additionally, the transcriptional regulation of autophagy might participate to biological processes that are regulated independently of the nutrient status of the cells, such as cellular differentiation and tissue development (Cinque et al., 2015). It will be important in the next years to understand whether different transcription factors regulate selective types of autophagy in a tissue and time specific fashion and if their modulation can be exploited for therapeutic purposes.

A selective modulation of autophagy might be beneficial for the treatment of several diseases for which there are no currently available therapies. Notably, several therapeutic benefits associated to administration of widely used drugs, such as aspirin and metformin, and food compounds, such as resveratrol and curcumin, might be due their ability to induce TFEB nuclear translocation and autophagy (Bao et al., 2016; Zhang et al., 2016; Wang et al., 2017; Yan et al., 2017; Chandra et al., 2018). Currently, whether these molecules can be repositioned for the treatment of genetic diseases is largely unexplored. Lastly, the use of computational approaches combined to an integrated analysis of omics data represents an invaluable tool to identify novel transcriptional modulators of autophagy (Napolitano F. et al., 2018).

## Author Contributions

CDM and CS wrote the manuscript. LC prepared the figure and wrote the figure legend.

## Conflict of Interest Statement

The authors declare that the research was conducted in the absence of any commercial or financial relationships that could be construed as a potential conflict of interest.
